# The Dynamic 3D Genome in Gametogenesis and Early Embryonic Development

**DOI:** 10.3390/cells8080788

**Published:** 2019-07-29

**Authors:** Feifei Li, Ziyang An, Zhihua Zhang

**Affiliations:** 1 CAS Key Laboratory of Genome Sciences and Information, Beijing Institute of Genomics, Chinese Academy of Sciences, Beijing 100101, China; 2 School of Life Science, University of Chinese Academy of Sciences, Beijing 100049, China

**Keywords:** chromatin structure, early embryonic development, gametogenesis, low-input Hi-C, single-cell Hi-C, formation mechanism of 3D genome

## Abstract

During gametogenesis and early embryonic development, the chromatin architecture changes dramatically, and both the transcriptomic and epigenomic landscape are comprehensively reprogrammed. Understanding these processes is the holy grail in developmental biology and a key step towards evolution. The 3D conformation of chromatin plays a central role in the organization and function of nuclei. Recently, the dynamics of chromatin structures have been profiled in many model and non-model systems, from insects to mammals, resulting in an interesting comparison. In this review, we first introduce the research methods of 3D chromatin structure with low-input material suitable for embryonic study. Then, the dynamics of 3D chromatin architectures during gametogenesis and early embryonic development is summarized and compared between species. Finally, we discuss the possible mechanisms for triggering the formation of genome 3D conformation in early development.

## 1. Introduction

As life begins, two terminally differentiated haploid gametes fuse into a diploid zygote followed by early embryonic development. Transcriptome and epigenome are both extensively reprogrammed during gametogenesis and early embryonic development. In gametogenesis, transcription stops at a defined point. DNA methylation and histone modifications are remodeled, although patterns are distinct in sperm and oocyte [1,2]. At a certain point after fertilization, transcription is activated (zygote genome activation, ZGA). This transfer is called the maternal-to-zygotic transition [3]. Recent studies also revealed dramatic reprogramming in the epigenomic landscape during this process [2,4]. The 3D structure of chromatin plays important roles in many nuclear processes, including transcription regulation and DNA replication [5,6]. In addition, the 3D genome is also highly correlated with the distribution of multiple epigenetic modifications [7,8]. These make gametogenesis and early embryonic development ideal models to investigate the relationship between transcription, epigenome, and chromatin structure, as well as the molecular mechanism underlying the formation of the 3D genome.

## 2. Low-Input Hi-C Methods and Analysis

Chromatin structure can be investigated by both conventional or super-resolution microscopy and chromatin conformation capture (3C)-based methods [9,10]. Especially, Hi-C is a widely used genome-wide conformation capture method. It measures all possible intra- and inter-chromosomal interactions [11,12]. However, traditional Hi-C methods require millions of cells. Investigation of chromatin structure in early embryonic development has been hindered by the rarity of embryonic material, especially for mammals. Thanks to the recent improvement of low-input, or even single-cell Hi-C, the reprogramming of chromatin structure during gametogenesis and early development was revealed.

Basically, the process of 3C and its derived methods can be simplified as follows: Crosslink, cut, label, ligation, enrichment, and quantification (Table 1) [13]. In an in situ Hi-C, the restriction enzyme-mediated DNA overhangs are filled in with biotin-labeled dNTPs, and then ligation is conducted within intact nuclei, improving the efficiency and resolution of the assay. This method was used to generate ultra-deep Hi-C maps for human cells with resolution up to 1 kb [11]. In 2017, two groups reported improved low-input Hi-C protocols according to in situ Hi-C, named optimized low-input in situ Hi-C and small-scale in situ Hi-C, respectively [14,15]. Both methods can generate high-quality Hi-C data using only hundreds of cells, and can accurately capture chromatin interaction patterns derived from millions of cells. The improvement mainly includes scaling down the reaction volume, reducing experimental procedures, minimizing tube exchanges and adding carrier RNA to avoid sample loss. For the first time, they utilized the optimized protocol to study the 3D chromatin architecture of mouse gametes and early embryos. Low-input Hi-C can also be used to analyze the primary tissues of patients and identify structural variations [16].

Although the starting material of low-input Hi-C is limited, the averaged ensemble structure of a cell population is still achieved. A few single-cell Hi-C methods have been developed to study the variability and dynamics of chromatin structure between single cells, and these have been used in the study of germ cells or early embryos (Table 1). Basically, these methods achieve single-cell analysis through the physical isolation of a single nucleus or the utilization of the combinatorial index to distinguish among different cells [17,18,19,20,21,22,23,24]. Single nuclei can be sorted before restriction enzyme digestion or after in situ ligation using microscopy or FACS [17,21]. The distinguishable index was introduced in the step of in situ ligation of restriction fragments and in the step of sequencing adaptor ligation [19]. The number of useful contacts and precision of the genome structures produced by these protocols differ greatly [25]. Generally, physical separation of single nuclei can get a larger number of effective ligations per cell, while combining indexes can analyze a relatively large number of cells with a single experiment. In addition, the library preparation is different between methods, and some omit the biotin enrichment step to improve chromatin retrieval. Single-cell Hi-C reveals a high degree of cell-to-cell variability and reveals the dynamics of chromosome structure during the cell cycle and some important biological processes, for instance, oocyte-to-zygote transition [22]. Additionally, Longzhi et al. developed Dip-C to reconstruct the genome structures of single diploid human cells, and revealed the distinct 3D structures of the maternal and paternal alleles [24].

The standard output of Hi-C is a contact matrix, including all pairwise interaction frequencies of any two loci at a given resolution [26]. The matrix has to be normalized carefully to remove bias in restriction length, GC content, and mappability [27]. A variety of tools have been developed for the analysis and visualization of Hi-C data, as reviewed previously [28,29]. These tools can be used to analyze low-input Hi-C data, as hundreds of millions of pairwise contacts can be detected. However, the data generated in a single-cell Hi-C experiment is ultra-sparse with, at most, hundreds of thousands of contacts per single cell [25]. We assessed the performance of current computational methods using ultra-sparse data at the single-cell level and found that most state-of-the-art methods do not work properly (manuscript in preparation). Thus, de novo detection of different layers of chromatin structure is difficult to perform in single cells. The analysis of single-cell Hi-C typically uses previously annotated features or the ensemble features as a reference and takes advantage of aggregate analysis to ask whether the same structure can be observed at the single-cell level. 

## 3. Hierarchical Organization of Interphase Chromatin

**Figure 1:**
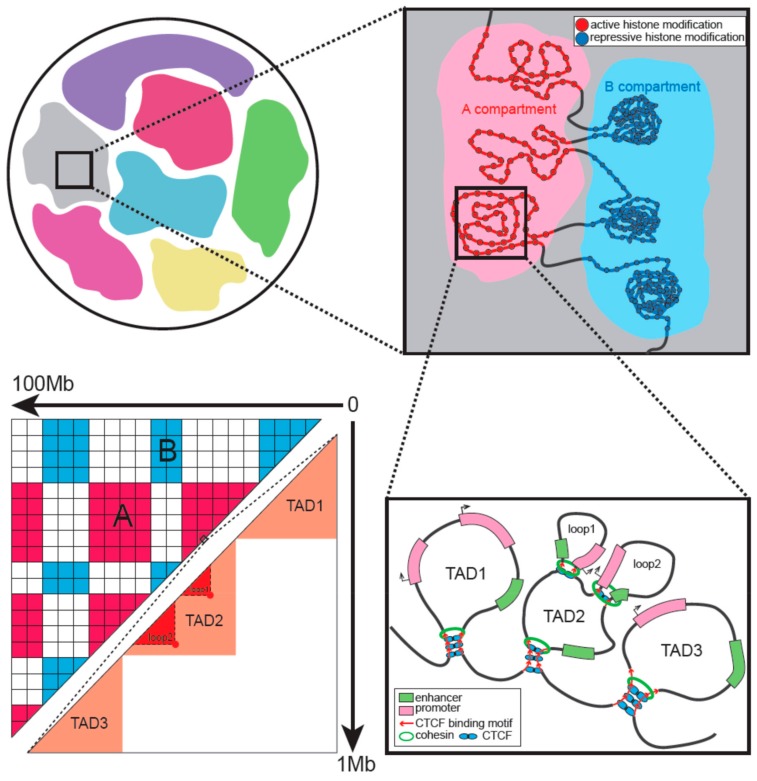
Hierarchical organization of interphase chromatin. Chromosomes occupy discrete space in the nucleus called chromosome territory. A and B compartments are characterized by active and repressive histone modifications, respectively. Topologically associating domains (TADs) and loops are formed by loop extrusion with the architectural proteins located in boundaries. The corresponding Hi-C heatmap is also illustrated. It shows the different scales of compartments and TADs.

Interphase chromatin in eukaryotic nuclei is folded into multiple layers of hierarchical structures, consisting of at least four levels from large to fine: Chromosome territories (CT), compartment A and B, topologically associating domains (TAD), and chromatin loops (Figure 1) [6,9,30]. Chromosomes occupy discrete space with limited, but appreciable, intermingling in the nucleus. This framework is called CT. Analysis of Hi-C data separates the CT into two compartments named compartment A and B. Compartment A is more closely associated with open, accessible, actively transcribed chromatin. Compartment B possesses converse characteristics and seems to be more densely packed. High-resolution Hi-C data revealed megabase-sized local chromatin interaction domains (TAD) as a pervasive structural feature. The domains are stable across different cell types and highly conserved across species. TADs also correspond to the physical and functional organization. Within TADs, looping interactions are the peaks on the contact matrices indicating regions that interact more frequently than flanking loci. Many loops involve regulatory interactions, such as promoter–enhancer interactions (Figure 1). Dynamic and cell-type-specific chromatin loops usually emerge during the construction of specific expression profiles.

Although chromatin conformation is relatively stable, it also needs to be flexible so that it can be re-organized. The above hierarchical 3D genome undergoes reprogramming during several processes, such as cell cycle, development, and stimulus-response [20,31,32,33]. It will be interesting to explore how chromatin is restructured during the production of gametes and early embryonic development in which expression and epigenome change dramatically.

## 4. Chromatin Remodeling in Gametogenesis and Early Embryonic Development

Chromatin remodeling has been studied at multiple time points during the process of early embryonic development in several animal models, including mouse, zebrafish, and *Drosophila*. Medaka was also investigated owing to the excellent assembly of its genome and easy accessibility of embryonic material. In general, the 3D genome is dramatically restructured in gametogenesis and early embryonic development. 

### 4.1. Chromatin Remodeling in Gametogenesis and Pre-Implantation Development in Mammals

Mice are the best-studied model in the study of 3D genome during gametogenesis and early embryonic development. The dynamics of chromatin structure have been investigated in the context of germ cell production, zygotic pronuclei, and pre-implantation development. 

**Figure 2:**
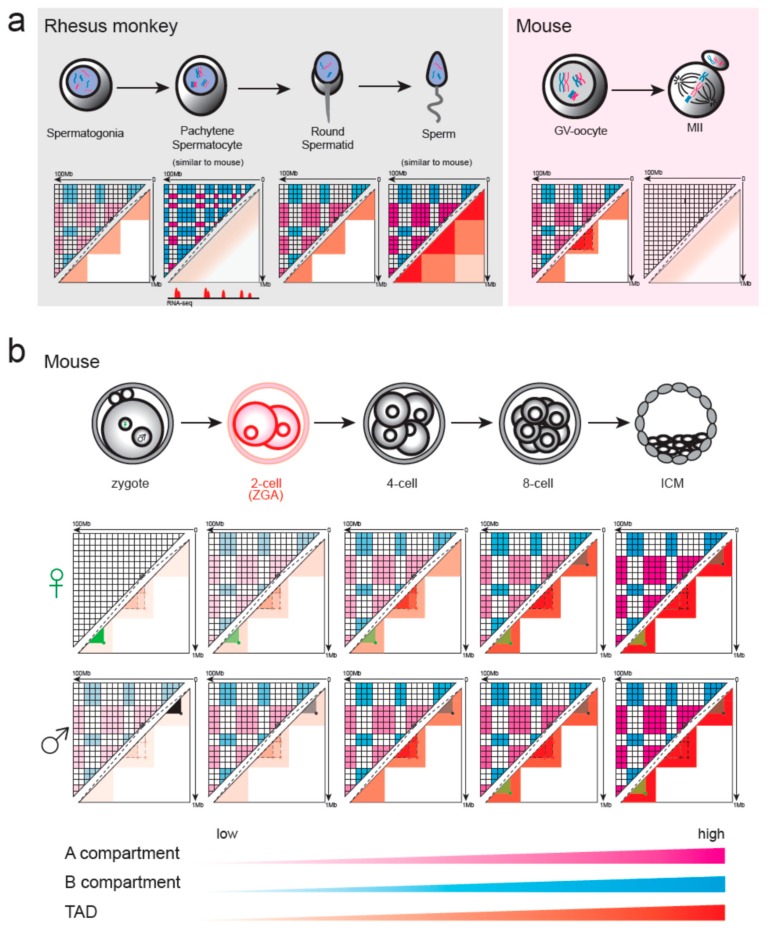
Chromatin remodeling in gametogenesis and pre-implantation development of mammals. The strength of compartments and TADs is illustrated with color bars with the darker color representing stronger structures. (**a**) Chromatin structure disappeared and was then reconstructed during rhesus monkey spermatogenesis. Pachytene spermatocyte had no conventional compartments A/B and TADs but showed a finer transcription-dependent compartment structure. Sperm showed extra-long-range interactions. Pachytene spermatocyte and mature sperm were also studied in the mouse and showed a pattern similar to that of the rhesus monkey. Germinal vesicle (GV) oocytes of the mouse had the typical higher-order structures, while MII had no such structures. (**b**) During mouse pre-implantation development, the strength of TADs, compartments, and loops is gradually enhanced. In the zygote, maternal nuclei had no compartmental structure although it is present in paternal nuclei. This strength difference of compartments between the two alleles continued until the 8-cell stage. Maternal-specific (green color) and paternal-specific (gray color) loops exist until the 8-cell stage at which time the loops converged. The period of zygote genome activation was colored in red.

In mammals, both male and female germ cells undergo meiosis, a process of one DNA replication followed by two cell divisions, resulting in the halving of the chromosome number. Chromatin structure during mouse spermatogenesis has been investigated in pachytene spermatocyte (PAC) and in mature sperm (Figure 2a) [34]. PAC, during which homologous chromosomes align and synaptonemal complex is formed, has a dramatically diminished TADs as shown in Hi-C. However, PAC displayed a highly refined transcription-correlated plaid pattern with high-transcription and low-transcription regions spatially segregated. Although the sperm nucleus has been classically considered as condensed with canonical histones exchanged for protamines, mouse sperm persists in the compartments and TADs structure in a manner similar to that of differentiated cells [14,15]. A unique characteristic of sperm chromatin is that it includes a high frequency of inter-chromosomal interactions and extra-long-range intra-chromosomal interactions, which may be associated with a smaller volume of sperm nuclei. In accordance with the layered structure, many transcription factors are associated with mouse sperm chromatin, including CTCF and cohesin [35]. In addition, sperm has promoters and enhancers in a primed state, which may guide future gene expression during embryogenesis; thus, sperm contains rich and complex epigenetic information [36].

In rhesus monkey, Hi-C analysis was done at more time-points during spermatogenesis (Figure 2a) [34]. TADs and compartments A/B dissolve and then reappear during sperm meiosis. These conventional structures exist in spermatogonia, although the signal is weak. In PAC, these canonical structures are strongly depleted but show highly refined transcription-correlated compartments as found in mice. In round spermatid and mature sperm, these layered structures re-establish and reinforce gradually. 

During fetal development, the oocyte enters meiosis and is arrested at the diplotene stage of prophase I, termed the germinal vesicle (GV) stage [37]. Analysis of 3D chromatin organization using single-nucleus Hi-C suggests that mouse GV oocytes show the typical higher-order structures, including loops, TADs, and compartments (Figure 2a) [22]. Single-nucleus Hi-C also reveals that the intensity of TADs, loops, and compartments significantly decreases from transcriptionally active immature oocytes to transcriptionally inactive mature oocytes [22]. After a hormonal surge, the oocyte continues meiosis and is arrested again in the metaphase of meiosis II (MII). Although chromatin accessibility at many putative CTCF binding sites in the MII-stage chromosomes is similar to that of the GV stage, low-input Hi-C shows that MII oocytes lack the typical interphase chromatin structure [14,15]. MII oocytes show a uniform interaction pattern along the whole chromosome, which is similar to the chromatin structure during mitosis [38]. It has been suggested that mitotic chromosomes are structured as helically arranged nested loop arrays formed by differential actions of condensin I and II [39]. Whether these proteins are also effective in forming MII stage chromatin and the 3D genome organization throughout the whole process of oocyte production need further investigation. 

After fertilization, the maternal genome inherited from the oocyte and the paternal genome provided by sperm co-exist as separate pronuclei in the zygote. In mouse zygotes, single-nucleus Hi-C was conducted in extracted maternal and paternal pronuclei (Figure 2b) [22]. Results show that TAD and loop structures are present at similar strengths in maternal and paternal nuclei by averaging over TADs and loops identified previously. However, maternal nuclei have no compartmental structure although it is present in paternal nuclei. Bulk Hi-C was also conducted in mouse zygotes, and unlike single-nucleus Hi-C results, TADs and distal chromatin interactions were obscure in both zygotes and two-cell embryos [14,15]. This discrepancy may be a result of different analytical methods, as described above. Bulk Hi-C de novo identifies structures, while single-cell Hi-C takes advantage of aggregate analysis to detect more ambiguous structures. Indeed, re-analyses of bulk Hi-C studies using aggregate-averaging analysis and normalized observed-over-expected maps support the presence of loops and TADs in two-cell embryos and even in the zygote, although the strength is weak [40]. The more poorly structured compartments in maternal allele compared with paternal allele are also observed in bulk Hi-C, and the difference continued as late as the 8-cell embryo stage [14]. In mouse, ZGA takes place in the 2-cell stage, and after ZGA, TADs and distal interactions gradually become more evident as development proceeds (Figure 2b). Intra-domain interactions between nearby regions increase first, and then interactions between distal regions within the domains increase later [14]. 

A recent study has shown that transcription factors (TFs) binding is persistent between gametes and early mouse embryos and that the long-range contacts they mediated are also trans-generationally inheritable (Figure 2b) [35]. Loops specific for maternal chromosome in zygote are present in the GV oocyte and are conserved in the maternal chromosomes throughout early embryonic development. These loops are not present in the paternal chromosomes until the 8-cell stage. The same is true for paternal-specific loops in zygote which present in sperm and persist in paternal chromosomes during development until the 8-cell stage when the maternal allele also produces the same loops. The mechanisms by which these allele-specific interactions are established and converge by the 8-cell stage are unknown. It is worth noting that differences of compartment strength between the two parental chromosomes also disappeared by the 8-cell stage. Crosstalk between paternal and maternal allele may be necessary for the convergence as spatial segregation of the parental genomes can be found as late as the 8-cell stage [14]. Dynamics of chromatin structure in parthenogenetic and androgenetic embryos may help answer these questions.

### 4.2. Chromatin Organization in Non-Mammalian Vertebrates during Early Embryonic Development

**Figure 3:**
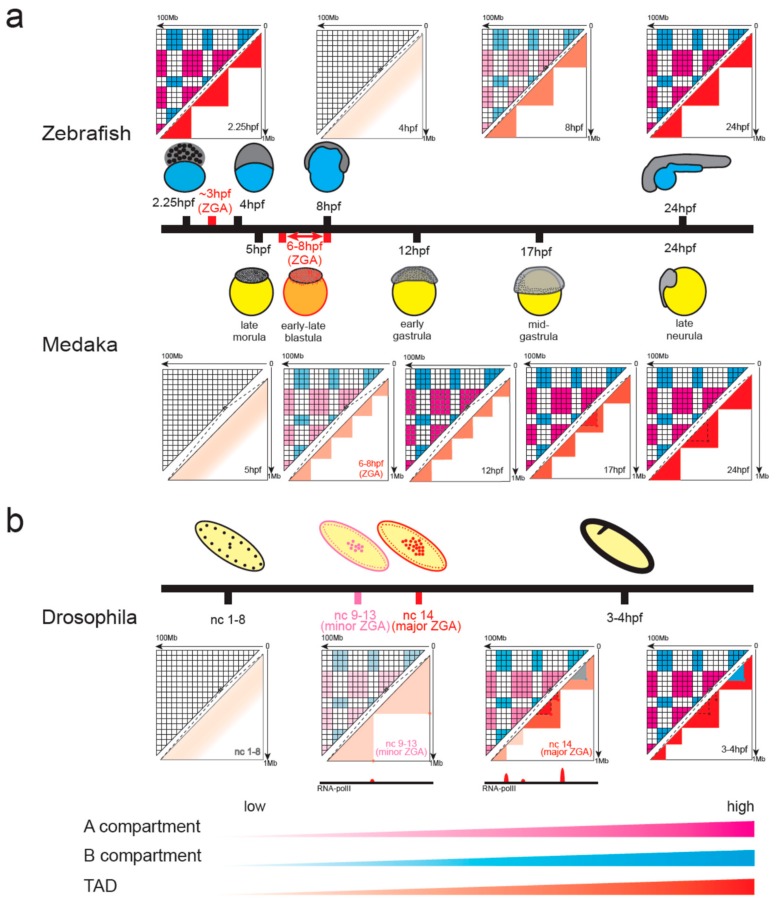
Chromatin remodeling in early embryonic development of zebrafish, medaka (**a**), and *Drosophila* (**b**). The period of zygote genome activation was colored in red. (**a**) Zebrafish chromatin displayed a unique pattern of systemic loss and regain. In medaka, chromatin structure was established in zygote genome activation (ZGA), but the size of TADs was small. Up to gastrulation, large contact domains matching the size of mature cells will form. (**b**) In *Drosophila*, chromatin architecture mainly emerges at the onset of ZGA, and TAD boundaries are established concomitant with the binding of RNA Pol II. Polycomb-dependent repressive loops (blue color) are only formed after midblastula transition.

In zebrafish development, chromatin structure displays a unique systemic loss and regain pattern (Figure 3a) [41]. Early in development, when there is no transcription, the genome is highly structured into TADs and A/B compartments. However, when the zygotic genome is activated in nuclear cycle 10 at about 3h post-fertilization (hpf), these organizations are lost. Later in development, the structured architecture re-establishes and increases gradually. 

In medaka, the establishment of chromatin structure takes place in ZGA (Figure 3a) [42]. Before ZGA, neither compartments nor domains are present in the 5 hpf sample. In the middle of ZGA, compartments and TADs begin to emerge accompanied by the formation of open chromatin. However, the size of the TADs is small and relatively ambiguous in the beginning. Up to gastrulation, large contact domains matching the size of mature cells will form. Additionally, despite consistent binding of CTCF throughout the whole embryonic development, interaction loops between CTCF-bound sites do not form until 17 hpf during gastrulation. 

### 4.3. Emergence of Chromatin Organization in Insect Embryos

In *Drosophila*, chromatin architecture also emerges at the onset of ZGA at nuclear cycle 14, and once established, most of the structure is stably maintained at later stages of development (Figure 3b) [43]. Prior to ZGA, the genome is mostly unstructured, displaying uniform contact probabilities across the whole genome, except for a few regions enriched in RNA Pol II binding and housekeeping genes that act as TAD boundaries. The compartments, TADs, and TAD boundaries become increasingly apparent during ZGA. TAD boundaries are established concomitant with the binding of RNA Pol II. At the end of embryogenesis in stage 16, TADs and compartments become even more pronounced. Active and repressive chromatin loops were identified during *Drosophila* development, but they were formed at different stages [44]. Zelda-dependent active loops are first formed during midblastula transition (cycle 9–13, minor ZGA), while polycomb-dependent repressive loops are formed after midblastula transition (Figure 3b). Repressive loops are important for embryo development by stabilizing gene repression. 

### 4.4. Similarities and Differences between Species

All species studied so far have experienced dramatic reconstruction of chromatin conformation during early embryonic development; however, the time point of establishment of 3D genome differed among species. In mouse, *Drosophila*, and medaka, the structured organization mainly emerged at the onset of ZGA. Once formed, most TADs stably maintained and became more evident at later stages of development in mouse and *Drosophila*. However, in medaka, the size of TAD is small in ZGA and large contact domains form until gastrulation. Zebrafish is specific for the appearance of a layered structure before ZGA. At the time of ZGA, the organization disappears and then re-establishes. Zebrafish and medaka are closer in evolution and experience a similar number of cell cycles before ZGA (about 10 cycles); however, the layered structure is present in zebrafish and absent in medaka at the beginning of embryo. Revealing this difference can help illustrate the formation mechanism for 3D genome. 

It is worth noting that the development rate is different among species. *Drosophila*, zebrafish, and medaka experience a period of rapid nuclear divisions after fertilization. All the chromatin reprogramming in zebrafish happens within the first 24 h post-fertilization at which time most organs have been established. Establishment of the 3D genome takes place at 2.5 hpf and 7 hpf for *Drosophila* and medaka, respectively. In mice, the first cell cycle occurs after 24 hpf. Although the time is longest for mice to form chromatin structure, it is the fastest when considering the cell cycles after fertilization. 

## 5. Mechanisms of 3D Genome Formation in Early Embryonic Development

### 5.1. Architectural Proteins

TADs and loops in mammals are thought to be formed by loop extrusion [45,46]. Two tethered cohesin-based loop-extruding factors slide in opposite directions and form progressive loops until they are hindered by convergent-oriented CTCF proteins (Figure 1). Loop extrusion explains the preferential orientation of CTCF motifs, enrichment of CTCFand cohesin at TAD boundaries, domain fusion upon boundary deletion, and the loss of TADs and loops in the conditional degradation of architectural proteins. Other proteins, such as YY1 and Znf143, are also present at CTCF sites and may regulate the specificity or frequency of chromatin interactions [47,48].

The function of architectural proteins in 3D genome reprogramming during embryonic development is emerging. In mouse sperm, the binding of CTCF and cohesin is maintained, although chromosomes are compacted. GV and MII oocytes also contain accessible sites corresponding to the CTCF motif [35,36]. This inheritable binding of architectural protein may ensure the construction of embryonic chromatin structure through loop extrusion. In agreement with this, conditional deletion of cohesin in the maternal allele of mouse zygote eliminated weak TADs and loops, while deletion of the cohesin release factor WAPL resulted in stronger structures [40]. TAD boundaries of zebrafish and medaka embryo also enrich convergent-oriented CTCF sites, suggesting that loop extrusion may also be responsible for TAD formation in these embryos [41,42,49]. Similar to mouse, CTCF binding was detected as early as 5 hpf before the emergence of structured chromatin in medaka, implying their function in instructing 3D genome formation. In *Drosophila*, other proteins may play a role analogous to that of CTCF/cohesin in mammals [50]. Motif analysis on open chromatin regions at TAD boundaries of *Drosophila* embryos has identified the enrichment of architectural proteins BEAF-32 and GAF, implying their roles in establishing insulation at TAD boundaries. In addition, Zelda-depleted embryos displayed a loss of insulation at strong Zelda-bound loci [43]. Zelda is a pioneering TF able to recognize its binding sites within the context of nucleosomes and establish accessible chromatin [51]. The importance of Zelda suggested that generation of a relaxed local chromatin environment may be necessary for the de novo establishment of chromatin conformation.

### 5.2. Transcription and Establishment of 3D Genome

Although architectural proteins play a role in early genome architecture, the stability of protein binding and absence of a layered structure in medaka early embryos before ZGA indicate that architectural proteins may not be sufficient for chromatin organization [35,42]. TAD boundaries are highly enriched in active, transcribed chromatin [26]. This suggests a possible connection between transcription and genome folding. Recent studies have shown that transcription can influence chromatin structure by affecting the binding of architecture proteins. In loop extrusion, some analysis implied that cohesin may be driven by transcription-induced supercoiling [52]. In both yeast and mouse, transcription can relocate cohesin over long distances on DNA [53,54]. Transcription readthrough could also remodel genome 3D structure [55]. In readthrough regions, elongating RNA polymerase II disrupts chromatin interactions by inducing cohesin displacement from CTCF sites, leading to locus decompaction. In addition, transcription can act as domain boundaries. A high-resolution Hi-C analysis of *Drosophila* revealed that small, transcriptionally active domains act as boundaries separating the silenced regions and that inhibition of transcription by inhibitors or heat shock affects the formation of compartmental domains [56]. 

In early embryonic development, structured organization mainly emerges at the onset of ZGA in mouse, *Drosophila*, and medaka. Especially, in *Drosophila*, insulation scores of newly established TAD boundaries are highly correlated with RNA Pol II binding strength supporting the insulation property of RNA Pol II (Figure 3b) [43]. In addition, loss of transcription leads to the loss of a boundary-like structure in transiently expressed genes. These synchronizations suggest that transcription can drive the construction of the 3D genome. However, in both mouse and *Drosophila*, transcription inhibition does not abolish the establishment of high-order genome structures [14,15,43]. In the presence of transcription inhibitors, ZGA was blocked, but TADs continued to consolidate in both species. In zebrafish, TADs and compartments can form in the absence of transcription before ZGA, also indicating that transcription is not a prerequisite for chromatin organization [41]. However, the strength of inter-TAD insulation was reduced by the lack of transcription in *Drosophila*. These data suggest that the establishment of high-order chromatin structures is independent of transcription in early embryo development; however, transcription might play a role in strengthening and maintaining chromatin conformation.

### 5.3. Transposable Elements (TEs) and 3D Genome Folding

A large proportion of the mammalian genome is comprised of TEs, and they are important to genome complexity and evolution variations [57,58]. In recent years, TEs have been associated with the 3D organization of chromatin [59]. On one hand, many TE families can act as cis-regulatory elements, such as enhancers and promoters. Enhancer-like repeats tend to interact with promoters and play roles in the organization of the 3D genomic structure [60,61]. Many candidate host genes were identified to be regulated by TEs, and pairwise interactions are also formed between TEs [62]. On the other hand, TEs often occur in domains or at domain boundaries [26,63,64]. Specific TE families were confirmed to have insulator function [65]. In addition, from an evolutionary perspective, activation of TEs can produce species-specific expansions of CTCF binding sites and influence chromatin structure [66].

A surge of TE transcription occurs during early embryonic development, contributing to the totipotency and activation of the genome [67]. This specific activation of TE may contribute to 3D genome establishment in early embryos. In the 2-cell mouse embryo after which the 3D genome emerges, TE has the highest transcription level and enriches in putative cis-regulatory sequences. These suggest TEs may contribute to the establishment of 3D genome [68]. A recent study revealed that the Murine Endogenous Retroviral Element (MERVL) family of TEs drives the 3D re-organization of the genome during early mouse embryogenesis [69]. By comparing the Hi-C contacts of totipotent-like 2-cell-like cells (2CLC) and mouse embryonic stem cells, the authors have shown that MERLV elements promote the formation of insulating domain boundaries in 2CLC. The formation of these boundaries is coupled to the upregulation of transcriptional activity and chromatin accessibility of MERLV loci. Artificial introduction of MERVL is accompanied by the formation of a domain boundary at the integration site. This analysis directly verifies a causal relationship between MERVL genomic location and chromatin structure. A similar local structural re-arrangement occurs at MERVL loci at the early 2-cell stage of mouse embryonic development. It should be noted that the establishment of domain boundaries coincides with active transcription at those loci; however, not all upregulated TEs attain insulator function, such as a subset of LINE1 types. This implies that the transcription of TEs is not sufficient for insulation and that other characteristics of MERVL are essential for the establishment of boundaries, all of which needs further investigation. 

### 5.4. Phase Separation

Growing evidence shows that the formation mechanisms of compartments and TADs/loops are different and even antagonistic. Although TADs and loops are globally lost through CTCF or cohesin depletion, compartments are unaffected and even reinforced [70,71,72]. On the contrary, knocking down cohesin release factor WAPL enlarges loop domains, but genome compartmentalization is weakened [73]. A striking difference of A and B compartment is the chromatin state, including active and repressive histone modifications, respectively [12]. Loss of cohesin, or its loading protein, reveals finer compartments matching epigenetic marks and local transcriptional activity better than wild-type Hi-C maps. This implies that compartments have a strong association with chromatin state [70,71]. Many studies have shown that liquid–liquid phase separation can drive the formation of nuclear subcompartments [74,75]. A growing amount of evidence points to a model that the association between compartmentalization and chromatin states were established through liquid–liquid phase separation. Actively transcribed domains have been shown to be phase-separated with super-enhancers [76]. Additional evidence came from in silico experiments, e.g., chromatin reprogramming in CTCF or cohesin deletion cells can be quantitatively reproduced by simultaneous action of loop extrusion and phase-separated compartmentalization [77]. However, direct evidence of the causal relationship between the chromatin states and compartmentalization remains lacking. 

During early embryonic development, the inconsistent presence of compartments and TADs or loops in the maternal chromosome of mouse zygote and in pachytene spermatocyte implied that the formation mechanism for these chromatin structures is also different (Figure 2) [22,34]. Much evidence has suggested that compartments of early embryos may also depend on phase separation of chromatin with different active states. Deletion of Scc1 eliminates loops or TADs in mouse zygote but reinforces the compartmentalization of active and inactive chromatin [40]. Pachytene spermatocyte also displayed a highly refined transcription-correlated plaid pattern, although TADs diminished. In-depth analysis of phase separation in early embryonic development will be valuable, considering the de novo establishment of A/B compartments and the widespread reprogramming of histone modifications during this process in many species [2]. In particular, histone modifications are asymmetric between maternal and paternal allele in early mouse embryos, and whether these asymmetries contribute to the difference of compartment strength in the two alleles is an open question.

## 6. Conclusions and Perspectives

With the help of improved low-input Hi-C and single-cell Hi-C, the dramatic reprogramming of the 3D genome during gametogenesis and early embryonic development has been revealed in several species. Differences exist in established pattern and speed between even closely evolutionarily related species. With more species being investigated, questions involving comparative developmental biology, e.g., the similarities and differences between mammals and non-mammals in the reprogramming of 3D genome during early development, will soon become addressable. The de novo establishment of well-organized genome 3D architecture in early embryonic development provides a wonderful model with which to study the mechanism underlying the formation of the 3D genome. In the coming years, more attention may be directed toward understanding the roles of basic biochemical or biophysical elements in this process. For example, phase separation has recently drawn much attention in explaining many cellular processes. It might also be critical in understanding the key events that trigger chromatin reprogramming. From the perspective of evolution, one interesting question that has been debated for centuries is whether epigenetic information can be transgenerationally inherited and whether such inheritance is stable. It is certain that the re-establishment of 3D genome architecture in gametogenesis and early embryonic development plays a central role in this information inheritance. Development of new technologies will help to extend research in this field, for example, multi-omics technology in single cells and super-resolution imaging in live cells.

## Figures and Tables

**Table 1:** Single-cell Hi-C techniques and characteristics.

Methods	Full Name	Procedure	Characteristics
Hi-C [11] (in situ Hi-C) [12]	Chromosome conformation capture by high-throughput sequencing	Crosslinking, restriction enzyme digestion, end filling with biotinylated dNTP and proximity ligation (ligation performed in intact nuclei in an in situ Hi-C), reverse crosslinking, sonication and streptavidin enrichment, and sequencing.	Widely used genome-wide method
Single-cell Hi-C [17,18]	Single-cell Hi-C	Similar to in situ Hi-C, individual nuclei selected using microscopy after proximity ligation. Remaining steps done in single cells separately. Sonication replaced with a second restriction enzyme to fragment ligation products.	The first single-cell chromatin structure method, relatively low throughput
Sci-Hi-C [19,20]	Single-cell combinatorial indexed Hi-C	Crosslinking, restriction digestion, distributed to 96 wells and barcoded bridge-adaptor ligation, nuclei pooled and proximity ligation, redistribution to 96 wells and barcoded sequencing-adaptor ligation, sequencing.	A larger number of single cells with fewer interactions per cell
Single-cell Hi-C [21]	Single-cell Hi-C	Crosslinking, single nuclei sorting with FACS, nuclei imaging, overlaid nuclei with low melting agarose. Remaining steps similar to in situ Hi-C but done in single cells.	Combination of imaging with determination of genome structure
Sn Hi-C [22]	Single-nucleus Hi-C	Similar to in situ Hi-C but omitting biotin incorporation. Single nuclei sorted by FACS after proximity ligation and then whole genome amplification was done to single nuclei.	More contacts per single cell
Improved multiplexed single-cell Hi-C [23]	Improved multiplexed single-cell Hi-C	Improved from [17], with flow cytometry sorting, Tn5 transposase library preparation, and an automation scheme.	Moderate contacts per single cell
Dip-C [24]	Single-cell Hi-C of diploid cells	Similar to Sn Hi-C [22]. Whole-genome amplification done with multiplex end-tagging amplification.	Distinguishes two haplotypes of each chromosome
